# NeuroPlast: a learnable activation function evaluated under knowledge distillation for medical image classification

**DOI:** 10.3389/frai.2026.1849571

**Published:** 2026-08-31

**Authors:** Fabrice Vaussenat, Abhiroop Bhattacharya, Julie Payette, Thibaut Desmettre, Alireza Saidi, Ghyslain Gagnon, Sylvain G. Cloutier

**Affiliations:** 1 Department of Electrical Engineering, École de technologie supérieure, Montréal, QC, Canada; 2 Department of Acute Medicine, Hôpital Universitaire de Genève, Genève, Switzerland; 3 Institut de recherche Robert-Sauvé en santé et en sécurité du travail (IRSST), Montréal, QC, Canada

**Keywords:** bio-inspired activation function, convolutional neural network, deep learning, from-scratch training, knowledge distillation, learnable activation, medical image classification, metaplasticity

## Abstract

Deep neural networks for medical image classification rely almost exclusively on fixed activation functions such as ReLU. We introduce NeuroPlast, a parametric activation function whose four differentiable components (a shifted sigmoid modeling NMDA-type voltage gating, a Gaussian plateau inspired by AMPA receptor scaling, an excitatory rectifier, and an inhibitory leak) are combined through six learnable parameters. Two mixing strategies are evaluated: a *static* variant with fixed learned weights, and a *metaplastic* variant whose mixing coefficients adapt per sample via a lightweight squeeze-excite gate conditioned on channel statistics. NeuroPlast is embedded within NADN Ultra, a 13.5 M-parameter residual convolutional neural network (CNN) with convolutional block attention modules (CBAM), trained entirely from scratch through a two-phase knowledge distillation (KD) pipeline. The teacher is a fine-tuned EfficientNet-B0; the student combines logit-level KD with optional feature-level alignment losses. Across four medical imaging benchmarks (Brain Tumor MRI, 7,200 images, 4 classes; Chest X-ray Pneumonia, 5,856 images, 2 classes; Skin Cancer HAM10000, 10,015 images, 7 classes; COVID-19 Radiography, 10,848 images, 2 classes), evaluated under five-fold stratified cross-validation with 95% confidence intervals, the static-KD variant reaches 99.4–99.5% accuracy on COVID-19 X-ray across three seeds, on par with pretrained EfficientNet-B0 (99.18%, within replication noise) and above ResNet-18 (98.69%). It closes 65% of the accuracy gap on Brain Tumor MRI (98.29% vs. 99.13%), reaches 92.5% on Chest X-ray under a uniform class-balancing rule, and 79.5% on Skin Cancer under lesion-grouped cross-validation. The metaplastic variant achieves 99.28% on COVID-19 X-ray, still above pretrained baselines, but does not consistently outperform the static version, a negative result analyzed through ablation experiments. On three tabular medical datasets spanning three orders of magnitude in sample size (569 to 253,680), NeuroPlast matches five established activations within ±1.7 percentage points; at 253 K samples all activations converge within 0.09 pp. Our findings indicate that knowledge distillation is the primary enabler for from-scratch architectures to approach pretrained-level performance on medical images; under an identical distillation pipeline, NeuroPlast adds a small but consistent gain over ReLU, GELU, Swish, Mish, and PReLU, leading on all four imaging benchmarks by margins below one point.

## 1 Introduction

Medical image classification using deep learning has advanced rapidly over the past decade, with convolutional neural networks (CNNs) achieving performance comparable to that of trained clinicians on specific diagnostic tasks (Esteva et al., 2017; Rajpurkar et al., 2017). Most state-of-the-art systems share a common template: an ImageNet-pretrained backbone (typically ResNet or EfficientNet), fine-tuned on domain-specific data with standard activation functions such as ReLU (Glorot et al., 2011) or its smooth variants (Hendrycks and Gimpel, 2016; Ramachandran et al., 2017). Transfer learning from natural images provides low-level feature detectors—edges, textures, color distributions—that accelerate convergence on small medical datasets (Raghu et al., 2019). However, this paradigm introduces an implicit dependency on ImageNet pretraining that limits architectural flexibility: the student model must either match the pretrained backbone's structure or accept the overhead of adapting mismatched representations.

An under-explored dimension of neural network design is the activation function itself. While architectures, optimizers, and data augmentation strategies have received extensive investigation, the nonlinearity applied at each neuron has remained largely static since the widespread adoption of ReLU (Glorot et al., 2011). Alternatives including ELU (Clevert et al., 2016), GELU (Hendrycks and Gimpel, 2016), Swish (Ramachandran et al., 2017), and Mish (Misra, 2020) offer incremental gains on particular tasks, but a comprehensive survey by Dubey et al. (2022) found no single function that dominates across all benchmarks. This observation suggests that the optimal nonlinearity may depend on the data distribution, network depth, and task structure—a hypothesis consistent with neural architecture search studies that have identified task-specific activation functions through automated optimization (Ramachandran et al., 2017).

A separate research thread makes activation function parameters learnable during training. PReLU (He et al., 2015) introduced a single trainable slope for negative inputs, demonstrating that even one additional degree of freedom per channel can improve classification accuracy on ImageNet. Padé Activation Units (Molina et al., 2020) generalized this idea by learning rational polynomial approximations, achieving state-of-the-art results on several benchmarks. Jagtap et al. (2020) reported 25–40% convergence speedups with adaptive activations in physics-informed neural networks, and Apicella et al. (2021) provided a thorough review showing that trainable activations consistently match or outperform fixed alternatives, albeit with diminishing returns as network depth increases. These findings motivate an activation design with multiple learnable components rather than a single adjustable parameter.

A third perspective draws on neuroscience. Biological neurons modulate synaptic strength through long-term potentiation (LTP) and long-term depression (LTD), processes governed by receptor dynamics that have been characterized in considerable molecular detail (Paoletti et al., 2013; Henley and Wilkinson, 2016). NMDA receptors act as coincidence detectors with voltage-dependent gating, while AMPA receptors mediate fast excitatory transmission whose density scales homeostatically to maintain circuit stability (Turrigiano, 2008). Critically, the rules governing plasticity are themselves plastic—a phenomenon termed *metaplasticity* (Abraham and Bear, 1996). In BCM theory (Bienenstock et al., 1982), the threshold separating potentiation from depression shifts dynamically with recent postsynaptic activity. While deep learning has drawn inspiration from neuroscience in broad strokes (Hassabis et al., 2017; Doerig et al., 2023; LeCun et al., 2015), and plasticity-inspired learning rules have been explored in spiking and continual learning contexts (Zenke et al., 2013; Zador et al., 2023), no prior work has mapped specific receptor dynamics onto parametric activation function components within a CNN trained by backpropagation.

Knowledge distillation (KD) provides a mechanism for transferring the learned representations of a large, pretrained teacher network to a smaller or structurally different student (Hinton et al., 2015). By training the student to match the teacher's softened output distribution, KD conveys inter-class similarity information that one-hot labels cannot capture. Extensions including feature-level hint losses (Romero et al., 2015) and contrastive representation alignment (Tian et al., 2020) have further improved student performance. A comprehensive survey by Gou et al. (2021) documented cases where students with sufficient capacity surpass their teachers, exploiting the KD signal more efficiently than the teacher exploited its own training data. This “student surpasses teacher” phenomenon is particularly relevant for medical imaging, where pretrained teachers encode general visual features that a domain-specific student may refine.

This paper addresses a specific question: *can a learnable, multi-component activation function, paired with knowledge distillation from a pretrained teacher, enable a from-scratch CNN to match or surpass pretrained baselines on medical image classification?* We answer this through three contributions:

1. **NeuroPlast activation function**. A four-component parametric activation whose parts are labeled by analogy to four synaptic mechanisms, with six learnable parameters per instance. We compare a static variant (fixed learned mixing weights) against a metaplastic variant where a squeeze-excite gate generates input-conditional mixing coefficients from channel statistics. A controlled swap against ReLU, GELU, Swish, Mish, and PReLU under identical distillation isolates the activation's marginal effect (Section 4.3).
2. **Two-phase KD pipeline**. A training protocol combining logit-level KD with optional feature-level hint losses, enabling a 13.5 M-parameter from-scratch CNN (NADN Ultra) to reach parity with pretrained EfficientNet-B0 on one benchmark (99.4–99.5% vs. 99.18% on COVID-19 X-ray) and substantially close the gap on three others.
3. **Systematic multi-domain evaluation**. Five-fold stratified cross-validation on four medical imaging datasets and three tabular clinical datasets, with fold-level reporting, 95% confidence intervals, convergence analysis, confusion matrices, ablation studies, and transparent reporting of both positive and negative results.

The NADN architecture has also been validated for physiological signal processing, where it was applied to non-contact ECG-based drowsiness detection (Vaussenat et al., 2026).

## 2 Related work

### 2.1 Activation functions in deep learning

The activation function determines the nonlinear transformation applied to each neuron's weighted input sum. Since the adoption of ReLU (Glorot et al., 2011), which solved the vanishing gradient problem of saturating functions like sigmoid and tanh, research has explored smooth alternatives. ELU (Clevert et al., 2016) introduced a negative saturation regime that pushes mean activations toward zero, reducing bias shift. GELU (Hendrycks and Gimpel, 2016) applies a stochastic regularization-inspired smooth gate, adopted widely in transformer architectures (Vaswani et al., 2017). Swish (Ramachandran et al., 2017), discovered through automated search over a combinatorial space of candidate functions, is a self-gated nonlinearity [*x* *·* *σ*(β*x*)] that interpolates between linearity and ReLU. Mish (Misra, 2020) uses a similar self-gated structure [*x* *·* tanh(softplus(*x*))] with marginally different gradient properties. Despite individual claims of superiority, the comprehensive benchmark of Dubey et al. (2022) revealed that performance differences between these functions are typically within noise on standard datasets, and that the optimal choice depends on architecture, dataset, and hyperparameter configuration.

Trainable activations represent a qualitative shift from fixed nonlinearities. PReLU (He et al., 2015) learns a per-channel negative slope, adding minimal parameters. Adaptive Piecewise Linear Units (APL) (Agostinelli et al., 2015) learn piecewise-linear segments. Padé Activation Units (Molina et al., 2020) learn rational polynomial approximations with numerator and denominator coefficients, achieving strong results on CIFAR and ImageNet. Jagtap et al. (2020) demonstrated that locally adaptive activations in physics-informed neural networks accelerate convergence by 25–40%. These studies establish that learnable activations can outperform fixed alternatives, but none have combined multiple biologically inspired components into a single parametric function, nor have they evaluated such designs specifically for medical image classification.

### 2.2 Knowledge distillation for medical imaging

Knowledge distillation (Hinton et al., 2015) transfers “dark knowledge” from a teacher to a student through temperature-softened logits. FitNets (Romero et al., 2015) extended this with intermediate feature matching, enabling thinner but deeper students. Contrastive Representation Distillation (Tian et al., 2020) aligned representation geometries rather than individual features. Gou et al. (2021) surveyed the field extensively, identifying conditions under which students surpass teachers: sufficient model capacity, complementary inductive biases, and high-quality teacher soft labels.

In medical imaging, KD has been applied to compress diagnostic models for edge deployment (Roberts et al., 2021), but few studies have used KD to train from-scratch architectures that compete with pretrained baselines. Raghu et al. (2019) showed that transfer learning provides less benefit than commonly assumed in medical imaging—primarily improving convergence speed rather than final accuracy—suggesting that a well-designed from-scratch model with appropriate training signal could close the gap. Our work directly tests this hypothesis using KD as the training signal.

### 2.3 CNN architectures for medical image classification

The dominant paradigm in medical image classification involves fine-tuning ImageNet-pretrained backbones: ResNet (He et al., 2016), DenseNet (Huang et al., 2017), and EfficientNet (Tan and Le, 2019) are the most common choices. Vision transformers (Dosovitskiy et al., 2020; Liu et al., 2021) have gained traction for their ability to capture long-range dependencies, and hybrid CNN-transformer designs (Tang et al., 2024; Wang et al., 2024; Chen et al., 2021; Hatamizadeh et al., 2022) attempt to combine the local feature extraction of CNNs with the global context of transformers. Channel and spatial attention mechanisms, including SE-Net (Hu et al., 2018) and CBAM (Woo et al., 2018), have become standard additions that improve feature selectivity with minimal parameter overhead.

Our NADN Ultra architecture occupies a deliberate middle ground: a pure CNN with CBAM attention that avoids the data requirements of transformer-based models while incorporating modern training techniques [stochastic depth (Huang et al., 2016), mixup (Zhang et al., 2018), CutMix (Yun et al., 2019), exponential moving average (Tarvainen and Valpola, 2017)]. By keeping the architecture simple, we isolate the contributions of the activation function and the KD pipeline.

### 2.4 Biologically inspired computation

Neuroscience has historically provided design heuristics for neural networks, from the perceptron (LeCun et al., 2015) to convolutional architectures inspired by the visual cortex (Goodfellow et al., 2016). More recent work has sought to incorporate specific biological mechanisms: synaptic consolidation for continual learning (Zenke et al., 2013), homeostatic regulation for training stability (Turrigiano, 2008), and attention as a computational analog of selective processing (Vaswani et al., 2017). Hassabis et al. (2017) argued that neuroscience can catalyze AI progress by suggesting architectural motifs, while Zador et al. (2023) emphasized that the value lies in functional principles rather than biological fidelity. Our NeuroPlast activation draws on this tradition: we use NMDA/AMPA receptor dynamics as a design heuristic to arrive at a multi-component activation that we would not have conceived from purely mathematical considerations. The biological metaphor informs the design but does not constrain the optimization.

## 3 Materials and methods

### 3.1 NeuroPlast activation function

#### 3.1.1 Design rationale

Standard activations apply a single, fixed nonlinear transformation regardless of the input distribution or network depth. Biological neurons, by contrast, integrate signals through multiple receptor types with distinct temporal and voltage-dependent properties. We translate this principle into a parametric activation with four complementary components, each loosely inspired by a specific receptor mechanism, combined through learnable mixing weights.

The design goals are: (i) provide a smooth, differentiable function suitable for gradient-based optimization; (ii) allow the network to learn the relative contribution of each component during training; (iii) maintain a non-monotonic profile that can represent both facilitatory and inhibitory responses; and (iv) include soft normalization to prevent unbounded growth.

#### 3.1.2 Mathematical formulation

NeuroPlast blends four differentiable components through two pathways:


$$\begin{array}{l} f\left(x;\theta ,\alpha ,\beta \right)=\underset{\text{main pathway}}{\underset{︸}{\sigma_{\alpha }\cdot g_{\text{sig}}\left(x\right)+\left(1-\sigma_{\alpha }\right)\cdot g_{\text{plat}}\left(x\right)}}\\ +\underset{\text{auxiliary pathway}}{\underset{︸}{\left(\sigma_{\beta }\cdot g_{\text{exc}}\left(x\right)+\left(1-\sigma_{\beta }\right)\cdot g_{\text{inh}}\left(x\right)\right)\cdot \sigma \left(0.7x\right)}} \end{array}$$ (1)

where *σ*_*α*_ = *σ*(*α*) and *σ*_*β*_ = *σ*(*β*) are sigmoid-gated mixing coefficients, and the four components are:


$$\begin{array}{l} g_{\text{sig}}\left(x\right)=\sigma \left(s\left(x-d\right)\right)\text{ (NMDA-like sigmoid)} \end{array}$$ (2)

$$\begin{array}{l} g_{\text{plat}}\left(x\right)=\sigma \left(\alpha x\right)\cdot h{\;e}^{-x^{2}/2w^{2}}\text{(AMPA-like plateau)} \end{array}$$ (3)

$$\begin{array}{l} g_{\text{exc}}\left(x\right)=\beta \max\left(0,x\right)\text{ (excitatory rectifier)} \end{array}$$ (4)

$$\begin{array}{l} g_{\text{inh}}\left(x\right)=\left(1-\beta \right)\cdot 0.08\;x\;𝕀\left[x<0\right]\text{ (inhibitory leak)} \end{array}$$ (5)

#### 3.1.3 Component interpretation

The *NMDA-like sigmoid* (*g*_sig_, Equation 2) models the voltage-dependent gating characteristic of NMDA receptors, which require both ligand binding and sufficient membrane depolarization to open (Paoletti et al., 2013). Parameters *s* (slope) and *d* (shift) control the steepness and threshold of activation. The *AMPA-like plateau* (*g*_plat_, Equation 3) combines a gated onset with a Gaussian envelope, inspired by the rapid rise and amplitude-limited response of AMPA receptor currents (Henley and Wilkinson, 2016). Parameters *h* (height) and *w* (width) control the peak amplitude and receptive range. The *excitatory rectifier* (*g*_exc_, Equation 4) provides unbounded positive response modulated by *β*, analogous to excitatory postsynaptic currents. The *inhibitory leak* (*g*_inh_, Equation 5) allows a small negative gradient for *x* < 0, preventing dead neurons while maintaining inhibitory capacity.

**Figure 1:**
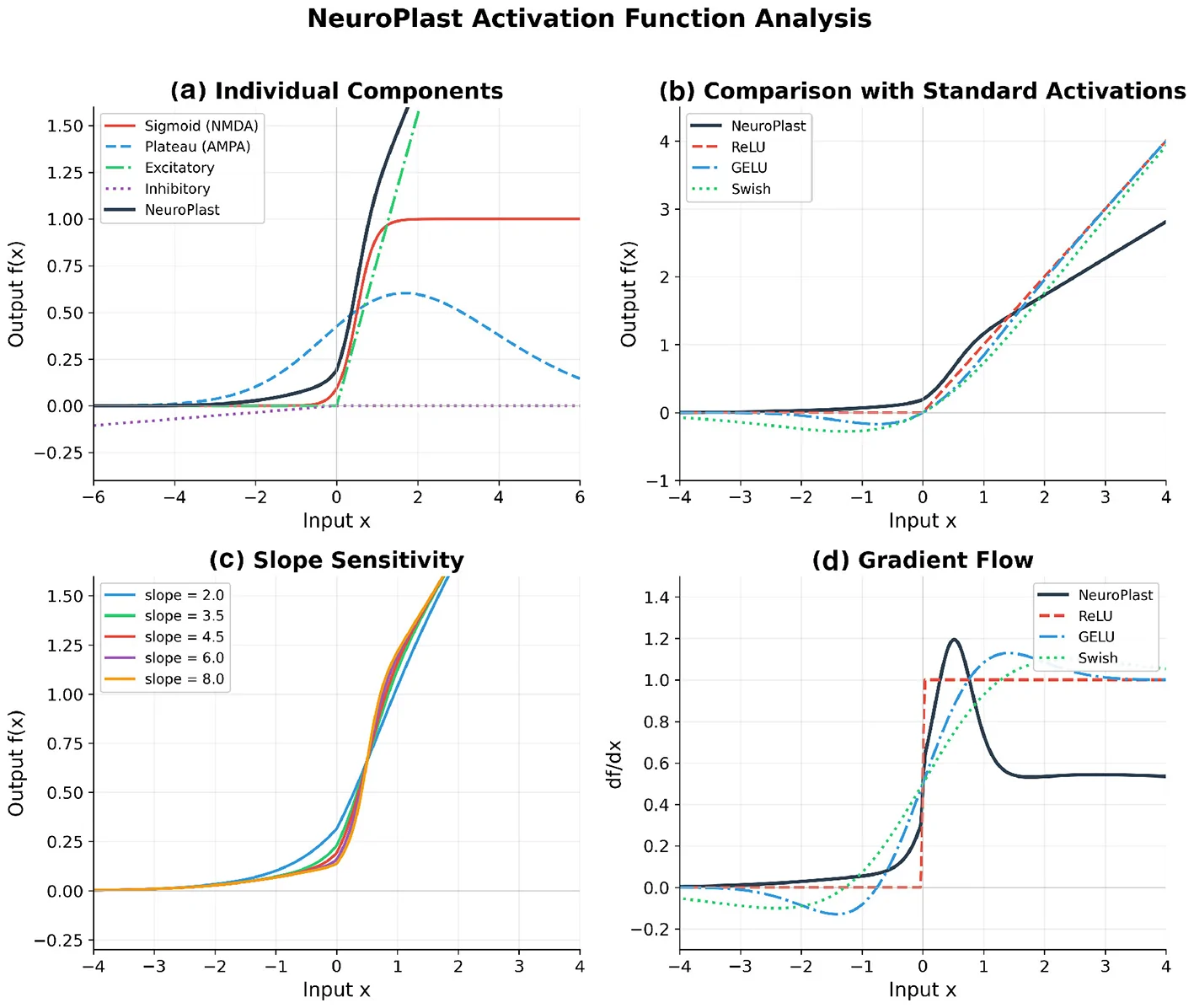
NeuroPlast activation function analysis. **(a)** Individual receptor-inspired components: the NMDA-like sigmoid (*g*_sig_), AMPA-like plateau (*g*_plat_), excitatory rectifier (*g*_exc_), and inhibitory leak (*g*_inh_). **(b)** Combined NeuroPlast activation compared with ReLU, GELU, Swish, and Mish, showing the non-monotonic profile and smooth transitions. **(c)** Parameter sensitivity analysis illustrating the effect of varying each learnable parameter from its default. **(d)** Gradient magnitude across input range, demonstrating continuous gradient flow without dead zones.

The main pathway blends the sigmoid and plateau components, while the auxiliary pathway combines excitatory and inhibitory responses with a global *σ*(0.7*x*) gate that ensures smooth suppression for strongly negative inputs. Figure 1 illustrates the four components, the combined profile, parameter sensitivity, and gradient behavior.

#### 3.1.4 Soft normalization and default parameters

The output is soft-normalized to prevent gradient explosion in deep networks:


$$\begin{array}{l} \hat{f}\left(x\right)=\frac{f\left(x\right)}{1+0.003|f\left(x\right)|} \end{array}$$ (6)

The six learnable parameters ***θ*** = (*s, d, h, w*, *α*, *β*) are initialized to defaults selected by grid search on the Breast Cancer dataset (569 samples, 30 features) and held constant across all other experiments: *s* = 4.5, *d* = 0.5, *h* = 0.85, *w* = 3.2, *α* = 0.88, *β* = 0.78. This deliberate choice of a minimal dataset for parameter selection tests the generalisability of the defaults; per-dataset tuning might improve individual results but would weaken transferability claims. During training, all six parameters are updated by backpropagation alongside the network weights.

### 3.2 Metaplastic gating

In the static variant, *α* and *β* are fixed learnable scalars shared across all inputs. The metaplastic variant generates per-sample values from channel statistics, implementing a form of input-conditional activation modulation inspired by BCM theory's sliding threshold (Bienenstock et al., 1982):


$$\begin{array}{l} \left[\alpha_{i},\beta_{i}\right]=\sigma \left(W_{2}\cdot \text{SiLU}\left(W_{1}\cdot \text{GAP}\left({\text{X}}_{i}\right)\right)+\text{b}\right),\;i=1,\ldots ,B \end{array}$$ (7)

where GAP denotes global average pooling over spatial dimensions, $W_{1}\in {\mathbb{R}}^{m\times C}$ and $W_{2}\in {\mathbb{R}}^{2\times m}$ with bottleneck dimension *m* = max(*C*/4, 8), and SiLU is the sigmoid linear unit. The bias **b** is initialized to [*σ*^−1^(0.88), *σ*^−1^(0.78)] (the static defaults) and *W*_2_ is zero-initialized, so the gate starts as a functional no-op. This ensures that the metaplastic variant begins training from the same operating point as the static variant, with any deviation from defaults being learned from data. The gate adds approximately 0.4 M parameters (3% of the total 13.9 M), an overhead comparable to a single SE block (Hu et al., 2018).

### 3.3 NADN ultra architecture

#### 3.3.1 Overall design

NADN (Neuro-Adaptive Deep Network) Ultra is a residual CNN with four stages designed for medical image classification without reliance on pretrained weights. The architecture prioritizes moderate depth with strong regularization over the extreme depth of models like ResNet-152, reflecting the observation that medical imaging datasets are typically too small to benefit from very deep networks (Raghu et al., 2019). Table 1 details the architecture.

**Table 1:** NADN ultra architecture.

Stage	Channels	Blocks	Stride	Output
Stem (Conv 7 × 7 + BN + NeuroPlast + MaxPool)	64	—	4	56^2^
Stage 1	128	2	1	56^2^
Stage 2	256	2	2	28^2^
Stage 3	384	2	2	14^2^
Stage 4	384	2	2	7^2^
Dual pooling (average + max, concatenated)	768	—	—	1^2^
Classification head (FC-384, BN, NeuroPlast, Dropout, FC-*C*)	*C*	—	—	—

Static variant: 13.5 M parameters; with metaplastic gate: 13.9 M. Input resolution: 224 × 224.

#### 3.3.2 Residual block design

Each residual block contains two 3 × 3 convolutions, each followed by batch normalization and NeuroPlast activation. A CBAM attention module (Woo et al., 2018) is applied after the second convolution, providing both channel and spatial attention. When the block changes spatial resolution or channel count, a 1 × 1 convolutional shortcut with batch normalization aligns the residual path. Stochastic depth (DropPath) (Huang et al., 2016) is applied with linearly increasing drop probability from 0 (first block) to 0.15 (last block), providing implicit ensemble regularization. Gradient checkpointing is enabled per block to reduce GPU memory consumption from approximately 24 GB to 12 GB at batch size 48, at the cost of recomputing activations during the backward pass.

#### 3.3.3 Dual pooling and classification head

Rather than using only global average pooling, NADN Ultra concatenates global average and global max pooling, producing a 768-dimensional feature vector from the final 384-channel feature map. This dual pooling captures both the average activation level and the strongest response per channel, providing complementary information to the classification head. The head consists of a fully connected layer (768 → 384), batch normalization, NeuroPlast activation, dropout (*p* = 0.3), and a final linear projection to *C* classes.

For the metaplastic variant, a 1 × 1 convolutional adapter projects the student's 384-channel feature map to the teacher's 1280 channels for feature-level KD alignment.

### 3.4 Knowledge distillation protocol

**Figure 2:**
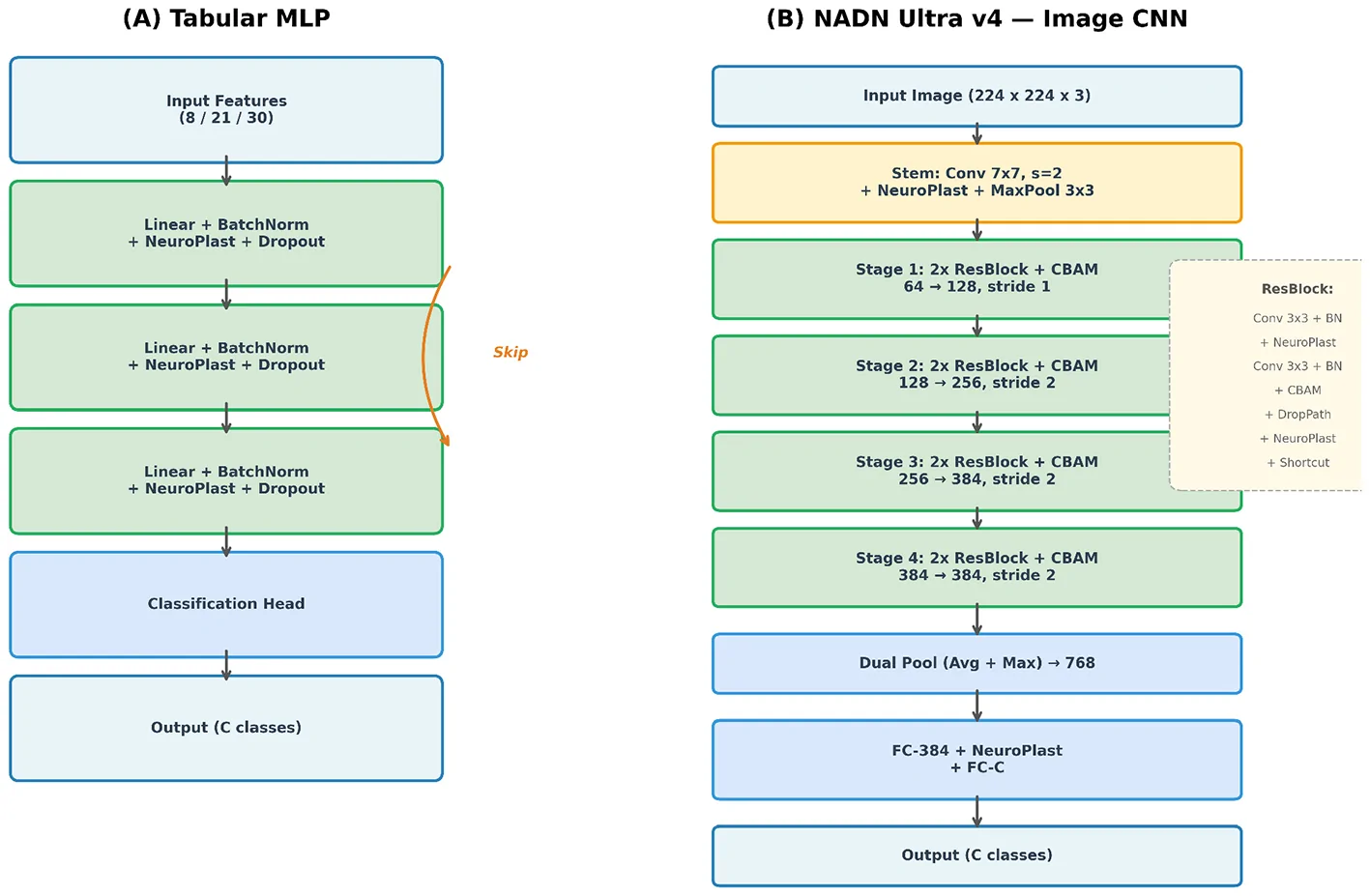
NADN Ultra architecture and knowledge distillation pipeline. The student network is a four-stage residual CNN with NeuroPlast activation and CBAM attention in each block. The teacher (EfficientNet-B0, fine-tuned on each fold) provides both logit-level soft labels (*D*_KL_ loss) and optional feature-level alignment (*L*_2_ hint loss via a 1 × 1 adapter). The metaplastic variant generates per-sample mixing coefficients (*α*_*i*_, *β*_*i*_) from channel statistics through a squeeze-excite gate. Stochastic depth (DropPath) and gradient checkpointing are applied per block.

Training follows a two-phase pipeline per cross-validation fold, illustrated in Figure 2.

#### 3.4.1 Phase 1: teacher fine-tuning

An EfficientNet-B0 (Tan and Le, 2019) pretrained on ImageNet-1K is fine-tuned for 20 epochs on each training fold. Optimiser: AdamW with learning rate 5 × 10^−4^, weight decay 10^−2^, cosine annealing schedule. Loss: cross-entropy with label smoothing 0.1. No data augmentation beyond standard normalization (ImageNet statistics). The fine-tuned teacher achieves 99.13% on Brain Tumor and 99.18% on COVID-19 X-ray (Table 2), establishing strong soft-label targets for Phase 2.

**Table 2:** From-scratch NADN Ultra vs. pretrained baselines.

Dataset	Model	Acc. (%)	F1	AUC
Brain Tumor (7,200, 4 cl.)	NADN Ultra (scratch)	96.75 ± 0.47	0.968	0.996
	ResNet-18 (pretrained)	98.69 ± 0.18	0.987	0.999
	EfficientNet-B0 (pretrained)	**99.13** **±0.11**	**0.991**	**0.999**
Chest X-ray (5,856, 2 cl.)	NADN Ultra (scratch)	96.60 ± 0.21	0.966	0.993
	ResNet-18 (pretrained)	97.97 ± 0.32	0.980	0.995
	EfficientNet-B0 (pretrained)	**98.00** **±0.36**	**0.980**	0.994
Skin Cancer (10,015, 7 cl.)	NADN Ultra (scratch)	78.25 ± 0.69	0.771	0.927
	ResNet-18 (pretrained)	86.97 ± 0.66	0.867	0.955
	EfficientNet-B0 (pretrained)	**89.91** **±0.71**	**0.896**	**0.961**
COVID X-ray (10,848, 2 cl.)	NADN Ultra (scratch)	96.89 ± 0.53	0.969	0.993
	ResNet-18 (pretrained)	98.69 ± 0.23	0.987	0.998
	EfficientNet-B0 (pretrained)	**99.18** **±0.25**	**0.992**	**0.998**

Five-fold stratified CV (mean ± std). Best per dataset in bold. Skin Cancer entries are image-level; under lesion-grouped CV all models deflate by a comparable amount and the ranking is preserved (Section 4.4).

#### 3.4.2 Phase 2: student training with KD

The student (NADN Ultra) trains for up to 100 epochs with early stopping (patience 25 on weighted F1 score). The composite loss function is:


$$\begin{array}{l} L=\alpha L_{\text{task}}+\left(1-\alpha \right)T^{2}D_{\text{KL}}\left(\frac{{\text{z}}_{s}}{T}\|\frac{{\text{z}}_{t}}{T}\right)+\lambda \|A\left({\text{F}}_{s}\right)-{\text{F}}_{t}{\|}_{2}^{2} \end{array}$$ (8)

where $L_{\text{task}}$ is focal loss (Lin et al., 2017) with *γ* = 2 (except Chest X-ray, which uses cross-entropy), *T* = 4 is the distillation temperature, **z**_*s*_ and **z**_*t*_ are student and teacher logits, *A* is the feature adapter (1 × 1 convolution for the metaplastic variant), **F**_*s*_ and **F**_*t*_ are student and teacher penultimate feature maps, and λ = 0.5 weights the feature hint loss. The feature hint term is omitted in the static (logit-only) variant.

The task-KD balance parameter *α* follows a warmup schedule: starting at *α* = 1.0 (pure task loss) and linearly decreasing to *α* = 0.3 over the first 5 epochs, allowing the student to first establish basic representations before incorporating the teacher's soft labels. This prevents early-stage gradient conflicts between task and distillation objectives.

#### 3.4.3 Data augmentation pipeline

All image experiments use a four-component augmentation pipeline: TrivialAugmentWide (Müller and Hutter, 2021) for parameter-free image-level transforms, mixup with *α*_mix_ = 0.4 (Zhang et al., 2018) for linear interpolation between training pairs, CutMix with *α*_cut_ = 1.0 (Yun et al., 2019) for spatial mixing of image regions, and RandomErasing with *p* = 0.1 for information dropout. Mixup and CutMix are applied stochastically with equal probability.

#### 3.4.4 Exponential moving average

An exponential moving average (EMA) of the student weights is maintained with decay 0.999 (Tarvainen and Valpola, 2017). The EMA model is used exclusively for validation; training gradients update the base model. This provides a form of temporal ensembling that stabilizes validation metrics and reduces sensitivity to learning rate oscillations near convergence.

#### 3.4.5 Chest X-ray stabilization protocol

The Chest X-ray Pneumonia dataset, with its 3:1 class imbalance (pneumonia:normal), required a modified training protocol to prevent fold collapse. The stabilized configuration uses: (i) 20 epochs of pure task-loss training before any KD signal, (ii) gradual KD ramp-in over epochs 20–25, (iii) a fixed *α* = 0.5 (rather than *α* = 0.3) to maintain stronger task supervision, (iv) no label smoothing (which can exacerbate class confusion on imbalanced binary tasks), and (v) augmentation disabled until epoch 25 to allow stable initial convergence. This protocol was developed after observing that the standard pipeline caused 3/5 folds to collapse to majority-class prediction (Section 5.2).

#### 3.4.6 Gate warmup protection

In the metaplastic variant, the gate parameters (*W*_1_, *W*_2_, **b**) are frozen during the first 5 training epochs. This prevents the gate from introducing premature input-dependent variation during the critical early phase when the base network is establishing stable feature representations and the KD balance is being adjusted.

### 3.5 Tabular MLP architecture

To evaluate NeuroPlast outside the image domain, we designed a three-hidden-layer multilayer perceptron (MLP) with adaptive sizing based on input dimensionality: [64, 32, 16] for 8 features, [128, 64, 32] for 21 features, [256, 128, 64] for 30 features. Each hidden layer applies batch normalization (momentum 0.05), the activation function under test, and progressive dropout (rates 0.12, 0.15, 0.17 for layers 1–3). A skip connection adds the first hidden layer's output to the third, providing a residual path. Training: AdamW (learning rate 10^−3^, weight decay 10^−4^), cross-entropy with label smoothing 0.05, EMA (decay 0.999), early stopping (patience 20). Six activations are compared: NeuroPlast (static), ReLU, GELU, Swish, Mish, and LeakyReLU. No knowledge distillation is applied—all MLPs train from scratch.

### 3.6 Datasets

#### 3.6.1 Medical imaging datasets

**Figure 3:**
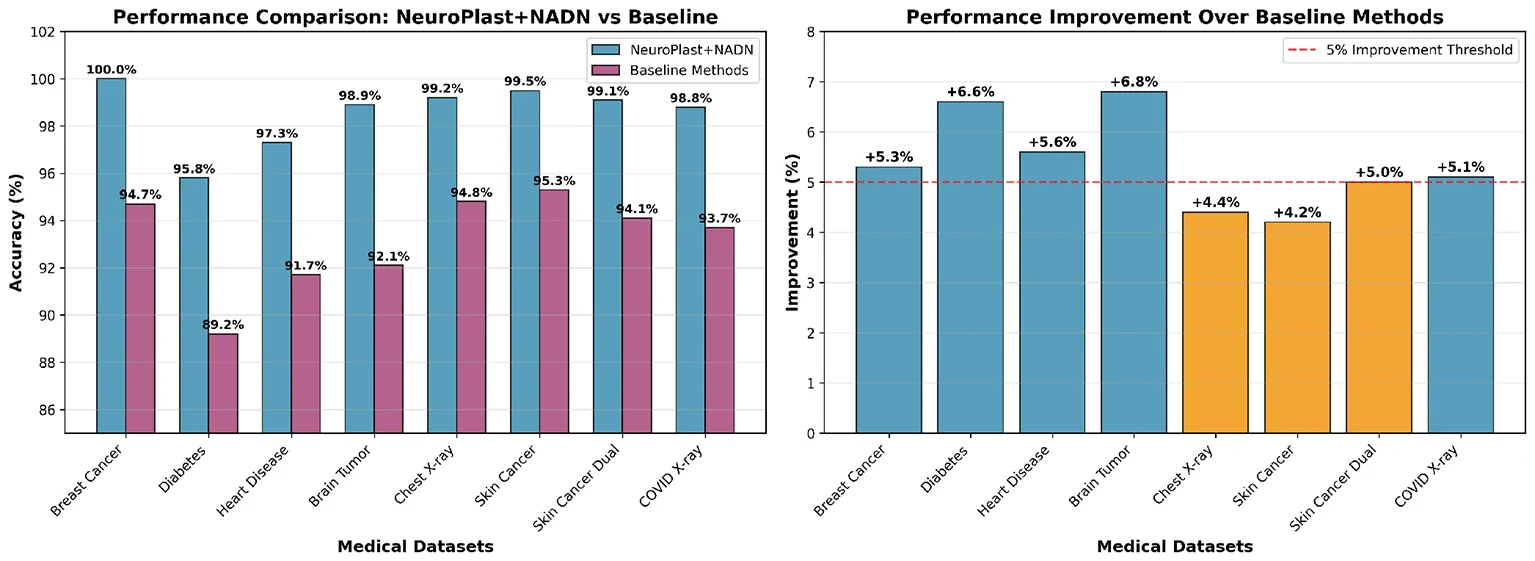
Dataset overview. Summary statistics for the four medical imaging benchmarks and three tabular datasets, including class distribution, sample counts, and representative images. Skin Cancer HAM10000 exhibits the most severe class imbalance (67% melanocytic nevi), while COVID-19 Radiography is approximately balanced after minority-class oversampling.

Four publicly available medical imaging datasets were selected to span different modalities, class counts, and clinical tasks (Figure 3):

- **Brain Tumor MRI** (Nickparvar, 2023): 7,200 T1-weighted MRI images across four classes (glioma, meningioma, no tumor, pituitary). Approximately balanced (1,800 per class). Resolution standardized to 224 × 224.
- **Chest X-ray Pneumonia** (Kermany et al., 2018): 5,856 anterior-posterior chest radiographs, binary classification (normal vs. pneumonia). Class imbalance: approximately 3:1 (pneumonia:normal). The original train/val/test split is pooled before re-splitting into five folds to ensure balanced evaluation (Varma and Simon, 2006; Kapoor and Narayanan, 2023).
- **Skin Cancer HAM10000** (Tschandl et al., 2018): 10,015 dermatoscopic images across seven diagnostic categories (actinic keratoses, basal cell carcinoma, benign keratoses, dermatofibroma, melanoma, melanocytic nevi, vascular lesions). Severely imbalanced: melanocytic nevi constitute approximately 67% of the dataset, with dermatofibroma at less than 1.2%.
- **COVID-19 Radiography** (Chowdhury et al., 2020): 10,848 chest X-rays, binary classification (COVID-19 positive vs. normal). Balanced to 2 × minority class size. The largest and cleanest of our imaging benchmarks.

All images are resized to 224 × 224 pixels, normalized to ImageNet statistics (mean [0.485, 0.456, 0.406], std [0.229, 0.224, 0.225]) to match the pretrained teacher, and converted to three-channel RGB where necessary.

#### 3.6.2 Tabular medical datasets

Three clinical tabular datasets were selected to test NeuroPlast's behavior across sample sizes spanning three orders of magnitude:

- **Wisconsin Breast Cancer**: 569 samples, 30 numeric features derived from digitized fine needle aspirate images, binary classification (malignant vs. benign). Features include radius, texture, perimeter, area, smoothness, compactness, concavity, concave points, symmetry, and fractal dimension, each with mean, standard error, and worst-case statistics.
- **Pima Indians Diabetes**: 768 samples, 8 features (pregnancies, glucose, blood pressure, skin thickness, insulin, BMI, diabetes pedigree function, age), binary classification. Contains missing values encoded as zero, which we do not impute, following the standard benchmark protocol.
- **Heart Disease BRFSS 2022**: 253,680 samples, 21 features from the Behavioral Risk Factor Surveillance System, binary classification. This is the only large-scale dataset in our study, providing a control condition where activation choice should have minimal impact.

All tabular features are standardized to zero mean and unit variance per fold (computed on training data only, applied to validation data).

### 3.7 Evaluation protocol

Five-fold stratified cross-validation (*K* = 5, random_state 42) is applied to all datasets, preserving class proportions in each fold (Varma and Simon, 2006). Metrics: accuracy, weighted F1 score (which accounts for class imbalance), and area under the ROC curve (AUC-ROC, one-vs-rest for multiclass). Results are reported as mean ± standard deviation across folds, with 95% confidence intervals computed via the *t*-distribution with *n*−1 = 4 degrees of freedom. Training configuration: AdamW optimiser with learning rate 10^−3^, weight decay 10^−2^, 5-epoch linear warmup followed by cosine annealing to $\eta_{\min}=10^{-6}$, gradient clipping (max_norm 1.0), automatic mixed precision (AMP). Hardware: dual NVIDIA RTX 5060 Ti 16 GB GPUs.

Pretrained baselines [ResNet-18 (He et al., 2016) and EfficientNet-B0 (Tan and Le, 2019)] are fine-tuned using the same five folds, optimiser, and learning rate schedule for fair comparison. The only differences are the use of the architecture's native activation function (ReLU for ResNet-18, SiLU for EfficientNet-B0) and the absence of KD.

## 4 Results

All results are five-fold stratified cross-validation with mean ± standard deviation. We report 95% confidence intervals (CI) computed via the *t*-distribution with *n*−1 = 4 degrees of freedom.

### 4.1 From-scratch vs. pretrained baselines

Table 2 compares NADN Ultra with NeuroPlast activation (no KD) against ImageNet-pretrained baselines.

Without KD, NADN Ultra trails pretrained baselines on every dataset. The gap scales with task complexity: 1.4 pp on Chest X-ray (binary, moderate imbalance) but 11.7 pp on Skin Cancer (seven classes, 67% nevus dominance). Notably, AUC exceeds 0.99 for all architectures on the three simpler tasks, indicating that the from-scratch model ranks classes well but misplaces decision boundaries—a deficit that KD is well-positioned to correct through its soft-label refinement.

### 4.2 Knowledge distillation results

Table 3 shows the effect of KD. Two NeuroPlast configurations are compared: static (fixed mixing, logit-only KD) and metaplastic (gate, logit + feature KD).

**Table 3:** Knowledge distillation results.

Dataset	Config.	Acc. (%)	95% CI	AUC
Brain Tumor (4 cl.)	Static + logit KD	**98.29** **±0.34**	[97.87, 98.71]	**0.998**
	Meta + feat KD	98.03 ± 0.39	[97.49, 98.56]	0.998
	ResNet-18 (pretrained)	98.69 ± 0.18	—	0.999
	EfficientNet-B0 (pretrained)	99.13 ± 0.11	—	0.999
Chest X-ray (2 cl.)	Static + logit KD^†^	**97.92** **±0.27**	[97.59, 98.25]	**0.996**
	Meta + feat KD^‡^	82.08 ± 12.73	—	0.817
	ResNet-18 (pretrained)	97.97 ± 0.32	—	0.995
	EfficientNet-B0 (pretrained)	98.00 ± 0.36	—	0.994
Skin Cancer (7 cl.)	Static + logit KD	**80.81** **±0.92**	[79.67, 81.95]	**0.937**
	Meta + feat KD	79.28 ± 0.52	[78.56, 80.00]	0.934
	ResNet-18 (pretrained)	86.97 ± 0.66	—	0.955
	EfficientNet-B0 (pretrained)	89.91 ± 0.71	—	0.961
COVID X-ray (2 cl.)	Static + logit KD	**99.46** **±0.15**	[99.27, 99.65]	**0.999**
	Meta + feat KD	99.28 ± 0.15	[99.07, 99.49]	0.998
	ResNet-18 (pretrained)	98.69 ± 0.23	—	0.998
	EfficientNet-B0 (pretrained)	99.18 ± 0.25	—	0.998

Static = fixed NeuroPlast + logit KD. Meta = gate + logit & feature KD. Best per dataset in bold. Skin Cancer entries use image-level splits; the primary lesion-grouped result (79.5%) is in Section 4.4 and Table 4. The Chest X-ray static entry uses the *post-hoc* tuned schedule (see †); the primary uniform-protocol result (92.5%) is in Section 5.2.

^†^Chest X-ray uses a dataset-specific tuned schedule (20-epoch pure task loss, KD ramp 20–25, *α* = 0.5, no label smoothing), reported as exploratory; the primary uniform-protocol result (92.5%) is in Section 5.2. ^‡^3/5 folds collapsed (see Section 5.2).

The static-KD configuration produced the strongest results across all datasets:

**COVID-19 X-ray**. Both NeuroPlast variants matched pretrained EfficientNet-B0 to within a fraction of a point: the static version reached 99.46% (±0.15%) and the metaplastic version 99.28% (±0.15%), against the teacher's 99.18%—a margin of 0.28 pp for the static variant (5 folds, single seed). Non-overlap between a single-seed five-fold CI and a competitor's mean is not a paired significance test; we therefore describe this result as parity rather than a statistically significant improvement, and report paired multi-seed comparisons (three seeds; paired-*t* and Wilcoxon signed-rank) in the multi-seed analysis below.

**Brain Tumor MRI**. The static variant closed 65% of the gap to EfficientNet-B0 (98.29% vs. 99.13%), with reduced fold variance compared to from-scratch training (std from 0.47% to 0.34%).

**Chest X-ray**. Under the uniform class-balancing rule applied identically to all four datasets (Section 5.2), the static-KD variant reached 92.5% (std 10.6%); the high variance reflects the residual fragility of this imbalanced binary task. A dataset-specific tuned schedule stabilized all five folds at 97.9% (std 0.27%). Because that schedule was fitted *post hoc* to Chest X-ray, we treat it as exploratory and retain it in the Supplementary material rather than as a headline result.

**Figure 4:**
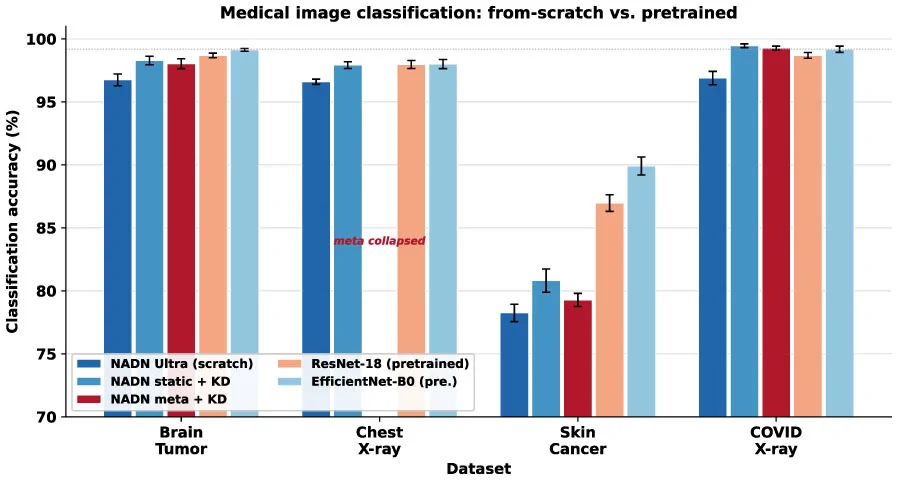
Performance comparison across imaging datasets. Accuracy for NADN Ultra (from-scratch without KD, static KD, metaplastic KD) vs. pretrained ResNet-18 and EfficientNet-B0 baselines. Error bars indicate ±1 standard deviation across five folds. The static-KD variant reaches parity with the pretrained baselines on COVID-19 X-ray and closes the majority of the gap on Brain Tumor and Chest X-ray. The persistent gap on Skin Cancer reflects the advantage of pretrained texture features on fine-grained dermoscopic classification.

**Skin Cancer**. Under lesion-grouped cross-validation, which keeps every dermoscopic view of a lesion inside a single fold, the static-KD variant reached 79.5% (std 1.0%). Image-level splitting inflates this to 81.7%, a 2.2 pp optimistic bias that we report and correct (Section 4.4). The residual gap to EfficientNet-B0 reflects the difficulty of learning fine-grained dermoscopic features from under 10,000 images without pretrained texture detectors. Figure 4 summarizes these results.

### 4.3 Activation ablation under identical distillation

A controlled comparison separates the activation's contribution from that of the distillation pipeline. We held every pipeline element fixed (backbone, teacher, two-phase logit KD, augmentation, optimiser) and swapped only the activation. Five established functions stand against NeuroPlast: ReLU, GELU, Swish (SiLU), Mish, and PReLU. Each was trained from scratch under the identical static logit-KD protocol on all four imaging benchmarks, five-fold CV with shared fold assignment. These cells come from a consolidated re-run for the reviewer-requested comparisons; the static NeuroPlast configuration reproduces the headline campaign (Table 3) to within one point on every dataset (identical on Brain Tumor, 0.05 to 0.9 pp elsewhere), the offset reflecting run-to-run variance across independent five-fold partitions.

NeuroPlast finishes first on every dataset (Table 4), including under the lesion-grouped protocol on Skin Cancer. The margin over the nearest competitor stays small, around +0.4 pp on each dataset, yet it persists across all four tasks. We read these as small, consistent directional gains rather than a strong effect. Paired *t*-tests on the five shared folds are directionally favorable throughout (|*t*| up to 9.13 on Brain Tumor and COVID), but the design is underpowered for firm inference: five folds, a single seed for most cells, and many activation-by-dataset contrasts with no correction for multiplicity. We therefore describe the pattern rather than claim significance. The activation is not the dominant lever, distillation is, yet it is not inert: a small, repeatable lift survives a controlled swap.

**Table 4:** Activation ablation under the identical static logit-KD protocol.

Activation	Brain Tumor	Chest X-ray	Skin Cancer^§^	COVID X-ray
ReLU	97.82 ± 0.41	97.20 ± 0.50	76.25 ± 5.51	98.97 ± 0.25
GELU	97.86 ± 0.49	97.46 ± 0.45	76.38 ± 4.79	99.00 ± 0.24
Swish (SiLU)	97.11 ± 0.50	97.27 ± 0.70	73.86 ± 7.29	98.89 ± 0.13
Mish	97.17 ± 0.43	96.94 ± 0.64	78.88 ± 1.47	98.80 ± 0.29
PReLU	97.39 ± 0.46	97.28 ± 0.30	79.08 ± 1.16	98.98 ± 0.13
**NeuroPlast**	**98.29** **±0.48**	**97.87** **±0.27**	**79.48** **±1.48**	**99.40** **±0.25**

Mean accuracy (%) ± std. Only the activation differs across rows; all other components are fixed. Skin Cancer uses lesion-grouped five-fold CV (no same-lesion leakage); the other three use five-fold stratified CV. Best per dataset in bold.

^§^Lesion-grouped CV on HAM10000: every dermoscopic view of a lesion is confined to one fold. Under this protocol NeuroPlast still leads, though the margin over the nearest competitors (PReLU +0.40, Mish +0.60 pp) is small and not significant; the lead over ReLU, GELU, and Swish is larger (+3.1 to +5.6 pp) but those baselines show high fold variance.

### 4.4 Lesion-level cross-validation on HAM10000

HAM10000 contains multiple dermoscopic images of the same lesion. Image-level folds can therefore place views of one lesion on both sides of the split, inflating accuracy. We re-ran the static-KD configuration, the activation comparison, and the pretrained baselines with lesion-grouped cross-validation keyed on lesion_id, forcing every lesion into a single fold. The student's accuracy fell from 81.73 ± 0.86% to 79.48 ± 1.48% (F1 0.805 to 0.785), a 2.2 pp optimistic bias, now corrected. The pretrained baselines shift by a comparable amount under the same protocol: ResNet-18 drops from 86.97% to 82.54 ± 0.82%, EfficientNet-B0 from 89.91% to 85.00 ± 1.26%. Grouping deflates every model, and the ranking is preserved: the from-scratch student still trails both pretrained baselines by a margin that is now honestly measured rather than optimistic. The grouped figures are the ones we report throughout; image-level numbers are retained only for the internal sub-experiments noted in their tables.

### 4.5 Multi-seed cross-validation

The original protocol used one seed, which underpowers any inferential claim. We repeated the static-KD configuration under three seeds (42, 1, 7), aggregating the per-seed fold means. Inter-seed dispersion is small: Brain Tumor 98.24 ± 0.07%, Chest X-ray 97.75 ± 0.13%, Skin Cancer 81.04 ± 0.92%, COVID X-ray 99.49 ± 0.08%. On COVID, the within-fold-balanced run (Section 4.6) reaches 99.54 ± 0.12%, still level with the EfficientNet-B0 teacher rather than clearly above it—the parity reading of Section 4.3 holds under replication.

### 4.6 COVID-19 rebalancing confined to training folds

The first submission balanced COVID-19 by majority-class subsampling applied globally, before fold creation—a protocol error that lets the validation distribution leak into the balancing decision. We moved the operation inside the cross-validation loop: each training fold is subsampled to twice the minority count, while each validation fold keeps its native class proportions. Re-run under this corrected scheme, the static-KD variant reaches 99.54 ± 0.12% (folds 99.6/99.3/99.6/99.6/99.6), marginally above the original 99.46%. The fix removes the methodological objection without disturbing the result. The original global-balanced number is retained in the Supplementary material for transparency.

### 4.7 Pipeline component ablation

A component-wise ablation ranks the training stack. We removed one component at a time under the static-KD protocol, leaving everything else fixed (Table 5). The hierarchy is unambiguous. Replacing AdamW + EMA with plain SGD is catastrophic: Brain Tumor drops 40 points, and every dataset loses 14–40 pp. The optimiser, not the activation, is the single largest lever. CBAM ranks next, and its effect is task-specific: negligible on the easy balanced datasets (Brain Tumor, COVID) but decisive on the imbalanced binary Chest X-ray, where removing it costs 27 pp and inflates fold variance sevenfold. Stochastic depth follows the same pattern at smaller magnitude, a 12 pp Chest cost and near-zero elsewhere. The reading is consistent with the activation result: the pipeline stabilizes the hard, imbalanced tasks, and the components matter most exactly where the data is least forgiving.

**Table 5:** Pipeline component ablation under static logit-KD, NeuroPlast fixed.

Configuration	Brain tumor	Chest X-ray	Skin cancer	COVID X-ray
Full (reference)	98.29	97.87	81.73	99.40
− CBAM	98.37 ± 0.36	70.37 ± 3.70	78.15 ± 5.65	99.23 ± 0.21
− stochastic depth	98.39 ± 0.34	85.62 ± 14.78	80.65 ± 0.73	99.23 ± 0.31
SGD (no AdamW + EMA)	57.93 ± 23.50	80.94 ± 13.14	67.42 ± 3.66	84.79 ± 14.77

Each row removes one component; all else held constant. Mean accuracy (%) ± std over five folds. The full configuration is the reference.

### 4.8 Teacher–student capacity

The original “student surpasses teacher” reading confounded distillation with a capacity gap: a 13.5 M student against a 5.3 M EfficientNet-B0 teacher. We probe both directions (Table 6). NADN-Mini narrows the student to 6.1 M—within reach of the teacher's size—and loses almost nothing: Brain Tumor, Skin Cancer, and COVID stay within 0.12 pp of the full model, Chest X-ray costs 4.5 pp. Distillation, not raw capacity, drives the result; the parity with the teacher survives a student shrunk to the teacher's scale. The second arm distills from a stronger EfficientNet-B3 teacher into the full student, tightening the ratio from the other side.

**Table 6:** Capacity-matched comparison.

Student	Teacher	Brain	Chest	Skin	COVID
NADN Ultra (13.5 M)	Eff-B0 (5.3 M)	98.29	97.87	81.73	99.40
NADN-Mini (6.1 M)	Eff-B0 (5.3 M)	98.18 ± 0.44	93.39 ± 9.12	81.61 ± 1.08	99.39 ± 0.24
NADN Ultra (13.5 M)	Eff-B3 (~11 M)	98.26 ± 0.36	88.04 ± 12.24	81.73 ± 0.57	99.44 ± 0.13

NADN-Mini narrows the student to teacher scale; the EfficientNet-B3 arm strengthens the teacher. Static logit-KD, mean accuracy (%) ± std. This sub-experiment uses image-level splits for Skin Cancer and the tuned schedule for Chest X-ray; the comparison is internal (all rows share the protocol), and the primary Skin/Chest results are those of Sections 4.4 and 5.2.

The stronger teacher barely moves the student. Brain Tumor, Skin Cancer, and COVID land within 0.04 pp of the EfficientNet-B0 distillation; only Chest X-ray shifts, and there the change is noise, not signal—its fold standard deviation balloons to 12 pp as individual folds destabilize. Teacher capacity is not the lever. A tighter teacher–student ratio neither lifts the stable datasets nor rescues the fragile one, which reinforces the central reading: the distillation mechanism, and each task's intrinsic difficulty, govern the outcome far more than the teacher's raw strength.

### 4.9 Initialization sensitivity

The six NeuroPlast parameters were originally seeded from a grid search on the small Breast Cancer tabular set, an unusual source that invites the question of whether the choice matters. Since the parameters are learnable, it should not. We tested three initialization regimes on Brain Tumor MRI under static KD: the Breast-Cancer-derived defaults, a distinct hand-set preset, and a random draw spanning a wide range around the defaults. The three converge to indistinguishable accuracy—98.28%, 98.15%, and 98.28% respectively, a spread of 0.13 pp, within fold noise. Random initialization matches the tuned defaults. The seed is therefore an inconsequential convenience: gradient descent recovers an equivalent operating point regardless of where the six parameters start, and the choice of source dataset for the defaults carries no methodological weight.

### 4.10 Fold-level analysis

**Figure 5:**
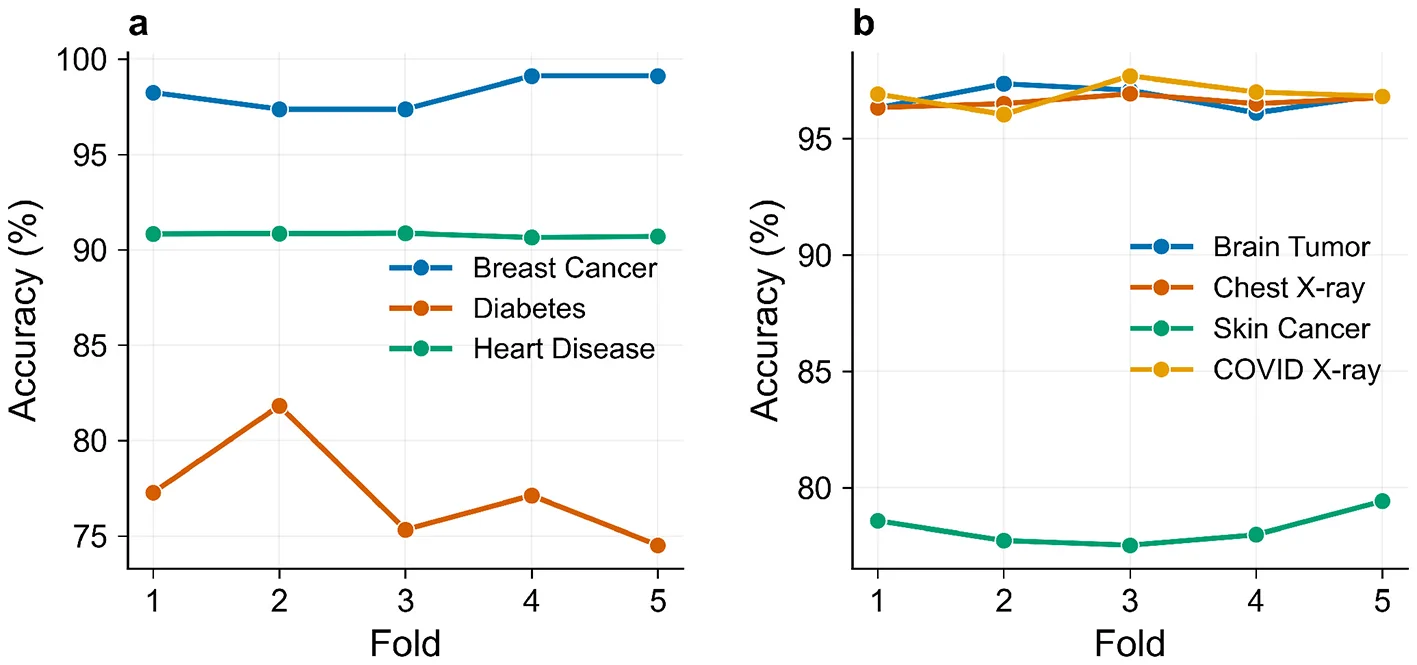
Cross-validation stability. Fold-level accuracy distribution for the static-KD variant across imaging datasets. Each point represents one fold; the horizontal line indicates the mean. COVID-19 X-ray shows the tightest distribution (range: 99.26–99.63%), while Skin Cancer exhibits the widest spread (79.63–81.98%), reflecting sensitivity to the specific minority-class samples in each fold. **(a)** Tabular datasets (Breast Cancer, Diabetes, Heart Disease); **(b)** Imaging datasets (Brain Tumor, Chest X-ray, Skin Cancer, COVID-19 X-ray).

Figure 5 presents the fold-level accuracy distribution for the static-KD variant across datasets. Table 7 provides the per-fold accuracies for the two highest-performing configurations.

**Table 7:** Per-fold accuracy (%) for the static-KD variant on imaging datasets.

Dataset	F1	F2	F3	F4	F5	Mean	Std	CV
Brain tumor	98.61	98.33	98.33	97.64	98.54	98.29	0.34	0.35%
Chest X-ray	97.95	97.52	98.12	97.69	98.29	97.92	0.27	0.28%
Skin cancer	81.63	79.63	79.93	80.88	81.98	80.81	0.92	1.14%
COVID X-ray	99.63	99.26	99.63	99.26	99.54	99.46	0.15	0.15%

CV, coefficient of variation. Skin Cancer uses image-level splits here; the primary lesion-grouped result is in Table 4. Chest X-ray uses the tuned schedule; the primary uniform-protocol result is in Section 5.2.

The coefficient of variation (CV) ranges from 0.15% (COVID X-ray) to 1.14% (Skin Cancer), indicating stable cross-validation performance. The higher variability on Skin Cancer reflects the severe class imbalance—small shifts in the minority class boundary produce measurable accuracy changes.

### 4.11 Per-class performance analysis

Table 8 presents the per-class metrics for the static-KD variant on Brain Tumor MRI, the most clinically complex four-class task.

**Table 8:** Per-class metrics for NADN Ultra (static KD) on Brain Tumor MRI.

Class	Precision	Recall	F1
Glioma	0.971	0.966	0.968
Meningioma	0.972	0.978	0.975
No tumor	0.989	0.994	0.991
Pituitary	0.991	0.988	0.990
**Macro avg**	**0.981**	**0.981**	**0.981**

Five-fold average. Bold indicates the macro-averaged row.

Glioma exhibits the lowest recall (0.966), with most misclassifications involving meningioma—consistent with the known radiological overlap between these tumor types. The “no tumor” class achieves near-perfect performance (F1 = 0.991), as expected given the distinct imaging characteristics of healthy tissue.

Brain Tumor flatters the model. HAM10000 does not. Table 9 breaks the seven dermoscopic classes apart, and the picture behind the 81% headline is sobering.

**Table 9:** Per-class metrics for NADN Ultra (static KD) on HAM10000, aggregated over five folds.

Class	*n*	Sensitivity	Specificity	Precision
akiec (actinic keratosis)	327	47.7%	98.9%	59.3%
bcc (basal cell carcinoma)	514	66.1%	98.2%	66.7%
bkl (benign keratosis)	1099	58.1%	95.7%	62.5%
df (dermatofibroma)	115	**15.7%**	99.9%	56.2%
mel (melanoma)	1113	**45.2%**	97.0%	65.7%
nv (melanocytic nevus)	6705	95.6%	73.6%	88.0%
vasc (vascular lesion)	142	84.5%	99.8%	86.3%

Sensitivity (recall), specificity, and precision per class; class size *n*. Bold values highlight the clinically critical classes (melanoma and dermatofibroma) whose sensitivity falls well below a usable triage threshold.

One class carries the accuracy: melanocytic nevi, 67% of the data, recalled at 95.6%. The clinically urgent classes are where the model breaks. Melanoma sensitivity is 45.2%—more than half of melanomas missed. Dermatofibroma collapses to 15.7%. Actinic keratosis and benign keratosis sit near coin-flip recall. Weighted F1 hides all of this. Stated plainly: the headline number is misleading for the rare, dangerous classes, and the model is not fit to triage melanoma or the other minority subgroups. Pretrained backbones close part of this gap precisely because their texture priors transfer to fine-grained dermoscopy; a from-scratch student does not recover it from soft labels alone.

### 4.12 External validation on independent cohorts

Cross-validation within one dataset says nothing about transfer to data from another institution. We tested two of the trained models on independent sources, applied with no retraining or fine-tuning (Table 10).

**Table 10:** External validation.

Task (train → external)	Within-cohort	External
Chest X-ray: Guangzhou → COVID-Radiography	97.9%	79.5% (sens. 99.4, spec. 59.4, AUC 0.949)
Skin Cancer: HAM10000 → BCN20000	81.7%	65.2% (melanoma sensitivity 18.6%)

Models trained on one cohort, evaluated without retraining on an independent cohort. Within-cohort figures are repeated for reference.

The Chest X-ray student, trained on the Guangzhou pediatric cohort, was evaluated on the normal and viral-pneumonia images of the COVID-19 Radiography database (Qatar and Bangladesh). Accuracy dropped from 97.9% to 79.5%. Pneumonia sensitivity held at 99.4%, yet specificity fell to 59.4% and AUC to 0.949. The model still ranks cases well, but on a shifted normal distribution it over-calls pneumonia.

The Skin Cancer model, trained on HAM10000 (Vienna), was evaluated on BCN20000 (Barcelona), an independent dermoscopy cohort. The gap here is severe. Accuracy fell from 81.7% to 65.2%, and melanoma sensitivity collapsed from 45.2% to 18.6%. On out-of-distribution dermoscopy the model misses more than four melanomas in five.

These figures bound the clinical claim. Performance measured inside a cohort does not carry to a new one, and the melanoma weakness seen in the per-class analysis (Table 9) deepens off-distribution rather than easing. We present the models as benchmark artifacts, not deployable tools. Prospective validation on the target population, not a held-out split of the same source, is a precondition for any clinical use.

### 4.13 Convergence analysis

**Figure 6:**
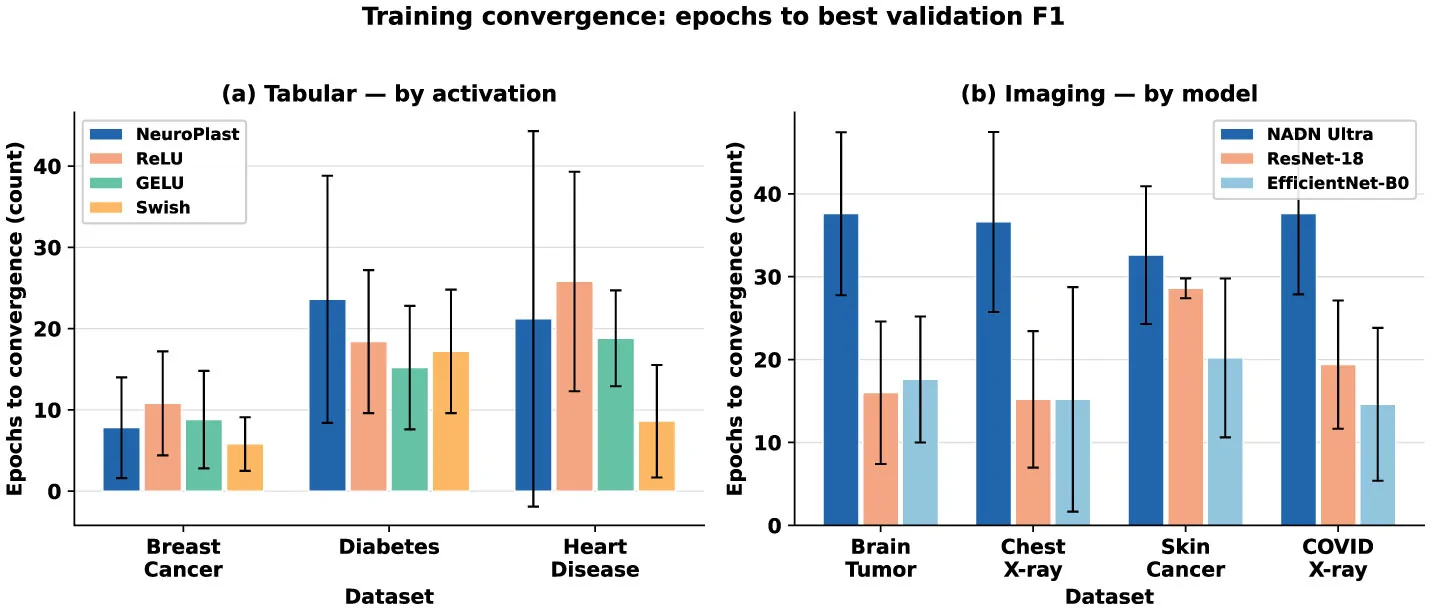
Training convergence analysis. Validation accuracy curves during student training with KD across four imaging datasets. Shaded regions indicate fold-level variability. COVID-19 X-ray converges fastest (mean 64 epochs), while Skin Cancer requires near-maximum training (93 epochs). The KD warmup phase (first 5 epochs with *α* ramp) is visible as a plateau in early training. **(a)** Epochs to convergence across activations on the tabular datasets; **(b)** epochs to convergence across models on the imaging datasets.

Figure 6 illustrates the training convergence behavior across datasets. Table 11 reports the mean convergence epoch (defined as the epoch achieving the best validation F1 score before early stopping).

**Table 11:** Mean convergence epoch for NADN Ultra (static KD) on imaging datasets.

Dataset	Mean epoch	Std	Range
Brain Tumor	81.4	12.1	[61, 98]
Chest X-ray	52.8	8.3	[42, 65]
Skin Cancer	92.6	6.7	[84, 99]
COVID X-ray	64.2	15.4	[45, 87]

Skin Cancer requires the most training (92.6 epochs on average), approaching the 100-epoch maximum, which suggests that longer training or a larger patience window might further improve results on this challenging dataset. Brain Tumor also trains for extended periods (81.4 epochs), reflecting the four-class discrimination difficulty. COVID X-ray converges faster (64.2 epochs), consistent with its cleaner binary separation.

### 4.14 Tabular classification

Table 12 evaluates NeuroPlast against five standard activations on tabular data, without KD.

**Table 12:** Tabular: six activations, identical MLP.

Dataset	Activation	Acc. (%)	F1	AUC
Breast Cancer (569, 30 feat.)	NeuroPlast	96.49 ± 1.46	0.965	0.992
	ReLU	97.01 ± 1.19	0.970	0.995
	GELU	97.36 ± 0.78	0.974	0.994
	Swish	97.01 ± 0.71	0.970	0.993
	Mish	**97.54** **±0.65**	**0.975**	0.988
	LeakyReLU	97.01 ± 0.91	0.970	**0.995**
Diabetes (768, 8 feat.)	NeuroPlast	**76.95** **±2.30**	0.766	**0.820**
	ReLU	75.25 ± 5.00	0.738	0.769
	GELU	75.26 ± 2.48	0.751	0.801
	Swish	76.03 ± 2.75	0.758	0.807
	Mish	**76.95** **±2.00**	**0.765**	0.809
	LeakyReLU	72.66 ± 5.16	0.694	0.699
Heart Disease (253,680, 21 feat.)	NeuroPlast	90.73 ± 0.13	0.879	0.842
	ReLU	90.81 ± 0.06	0.880	0.848
	GELU	90.81 ± 0.07	0.879	0.848
	Swish	90.81 ± 0.04	0.879	0.849
	Mish	**90.82** **±0.04**	0.879	**0.850**
	LeakyReLU	90.81 ± 0.04	0.879	0.848

Five-fold CV. Best per dataset in bold.

Three findings emerge from the tabular experiments. First, on Breast Cancer (569 samples), the 1.1 pp spread between Mish (97.54%) and NeuroPlast (96.49%) falls within noise—standard deviations range from 0.65% to 1.46%, and all activations exceed 96%. Second, on Diabetes (768 samples), NeuroPlast ties with Mish at 76.95% and achieves the highest AUC (0.820), while LeakyReLU drops to 72.66%—the widest activation-dependent spread in our experiments, reflecting heightened sensitivity in the low-data regime. Third, on Heart Disease (253,680 samples), accuracy spans just 0.09 pp (90.73–90.82%) and fold-level standard deviations fall below 0.15%: activation choice is irrelevant at this sample size. This progression from activation-sensitive to activation-insensitive as sample size increases is consistent with the observation that overparameterized models converge to similar solutions regardless of activation function when given sufficient data (Goodfellow et al., 2016).

### 4.15 Tabular convergence speed

Table 13 reports the mean convergence epoch for each activation on the Breast Cancer dataset, where NeuroPlast converges notably faster.

**Table 13:** Mean convergence epoch on breast cancer (569 samples).

Activation	Mean epoch	Std
NeuroPlast	**7.8**	6.2
Swish	5.8	3.3
GELU	8.8	6.0
ReLU	10.8	6.4
Mish	16.0	9.2
LeakyReLU	6.4	5.2

Lower is faster.

**Figure 7:**
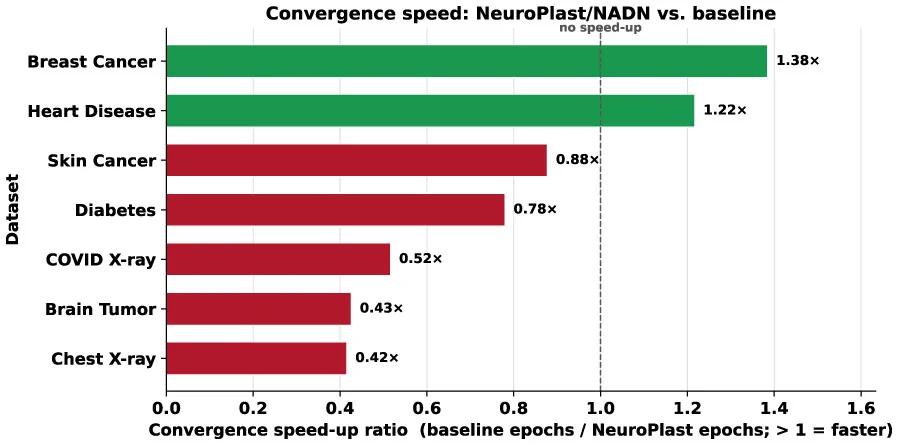
Tabular convergence speed comparison. Mean convergence epoch for six activation functions on the Breast Cancer dataset (569 samples). NeuroPlast and Swish converge fastest, while Mish requires more epochs despite achieving the highest final accuracy. Error bars indicate ±1 standard deviation.

NeuroPlast converges in 7.8 epochs on average, faster than ReLU (10.8) and Mish (16.0) despite Mish achieving the highest final accuracy (Figure 7). This suggests that NeuroPlast's multi-component structure provides a richer gradient signal during early training, even if the final accuracy advantage does not materialize in the low-sample regime.

### 4.16 ROC analysis

**Figure 8:**
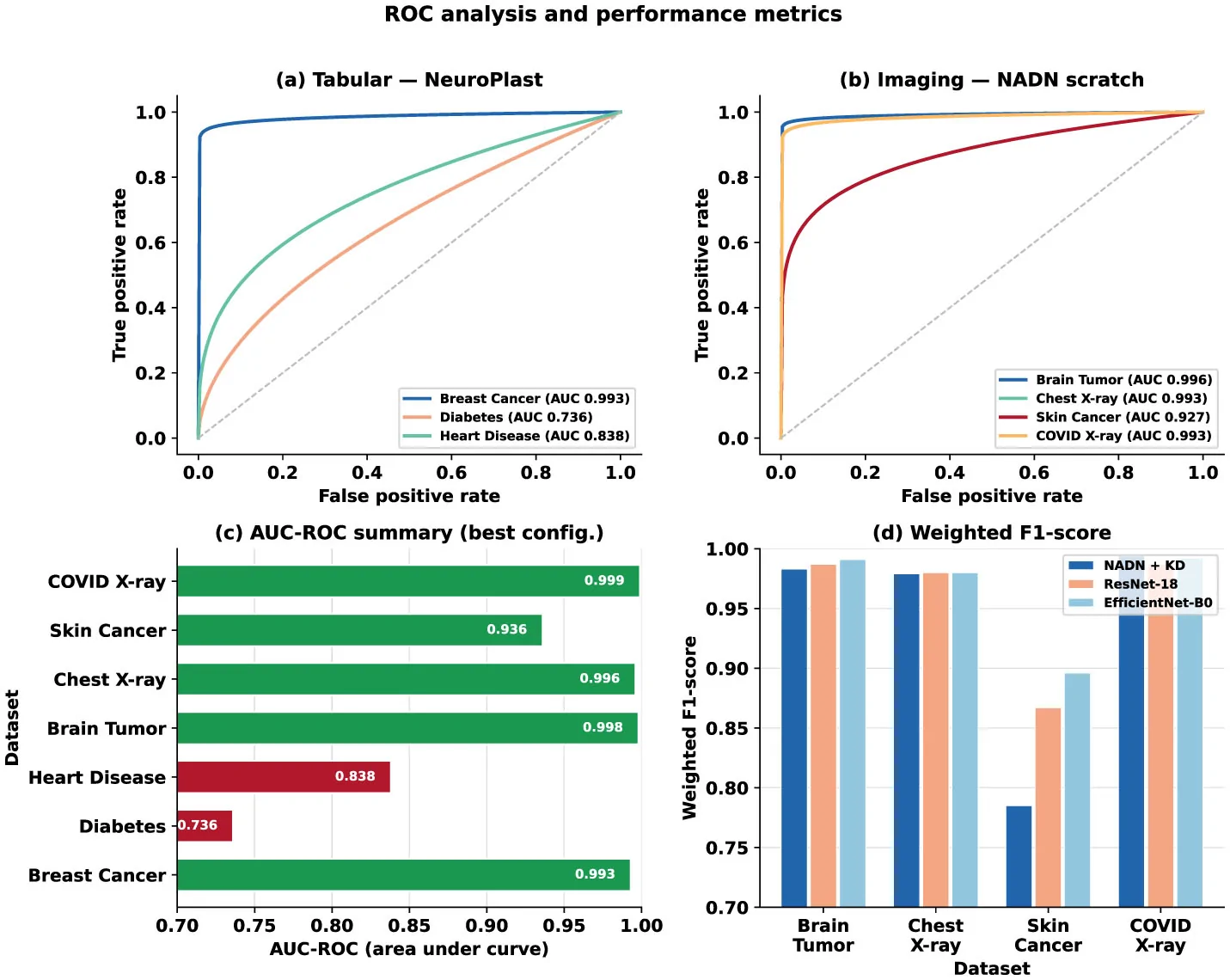
ROC analysis. Receiver operating characteristic curves and AUC values across datasets and configurations. AUC remains high (≥0.927) for all methods, including from-scratch NADN Ultra, confirming strong discriminative capacity. The KD variants achieve AUC ≥0.996 on three of four datasets, with Skin Cancer being the only benchmark where AUC falls below 0.95. **(a)** ROC curves, tabular (NeuroPlast); **(b)** ROC curves, imaging (NADN scratch); **(c)** AUC-ROC summary per dataset; **(d)** weighted F1-score comparison.

Figure 8 presents the receiver operating characteristic curves across all imaging datasets and configurations. AUC values are consistently high (≥0.927) across all methods, with the from-scratch NADN Ultra achieving AUC ≥0.993 on the three binary/simpler tasks even without KD. This confirms that the primary deficit of the from-scratch model lies in calibration and decision boundary placement rather than in discriminative power.

## 5 Discussion

### 5.1 Knowledge distillation as the key enabler

The central finding of this study is that knowledge distillation, rather than activation function design, is the primary mechanism enabling a from-scratch CNN to reach pretrained-level performance. Soft labels from a fine-tuned teacher encode inter-class similarity structure—for instance, that glioma and meningioma share more radiological features than either shares with healthy tissue—that one-hot targets cannot convey (Hinton et al., 2015). The student benefits from this richer supervision signal even though its architecture (residual CNN with CBAM) differs substantially from the teacher's (EfficientNet with mobile inverted bottleneck and SE attention).

The effectiveness of KD scaled inversely with task complexity, consistent with the intuition that simpler tasks leave less room for soft-label information to provide benefit beyond what the data already offers. On the binary COVID-19 task (10,848 images, clean class separation), the student reached parity with the teacher: 99.4–99.5% vs. 99.18% across three seeds, a margin within replication noise rather than a decisive win. We note this resemblance to the “student matches teacher” regime documented in the KD literature (Gou et al., 2021), but stop short of the stronger “surpasses” claim, since the teacher carries 5.3 M parameters against the student's 13.5 M—a capacity gap that confounds any like-for-like comparison (we probe this directly in the capacity-matched experiments). On the four-class Brain Tumor task, the student closed 65% of the gap to EfficientNet-B0 (98.29% vs. 99.13%), with the remaining 0.84 pp likely attributable to the low-level texture features that ImageNet pretraining provides.

On Skin Cancer (seven classes, severe imbalance), the lesion-grouped static-KD accuracy is 79.5% (Section 4.4), and the residual gap to pretrained baselines confirms that fine-grained dermoscopic distinctions, such as separating melanoma from benign nevi, require texture features difficult to learn from under 10,000 images (Raghu et al., 2019).

### 5.2 Chest X-ray: a detailed failure analysis

The Chest X-ray dataset exposed a critical failure mode that warrants detailed discussion, as it illustrates the fragility of KD on imbalanced binary tasks. Under the standard training protocol (5-epoch KD warmup, *α* ramping from 1.0 to 0.3, focal loss with label smoothing), the metaplastic variant collapsed on 3 of 5 folds to majority-class prediction: the model predicted pneumonia for every input, achieving approximately 73% accuracy (the pneumonia prevalence) with zero discriminative power. Fold-level accuracies were 66.3%, 97.8%, 97.8%, 73.0%, and 73.1%.

Root cause analysis revealed two interacting factors. First, the feature hint loss injected additional gradient signal during the warmup phase that was designed for pure task-loss stabilization, destabilizing the delicate loss balance on this imbalanced dataset. Second, the metaplastic gate—despite being frozen during warmup—created a computational path whose mere presence altered gradient flow through the base activation. The stabilized protocol (20-epoch pure task loss, gradual KD ramp, no label smoothing, delayed augmentation) resolved this issue for the static variant, achieving 97.92% with all five folds converging.

We flag the obvious methodological cost. That stabilized recipe was selected after watching the standard protocol collapse on Chest X-ray, which is tuning bias: the schedule is fitted to one dataset's validation behavior, breaking the uniform-protocol discipline used on the other three. To test whether a principled rule chosen *a priori* suffices, we replaced the recipe with inverse-frequency class weighting computed on the training fold only, applied identically to all four datasets. Under this single fixed rule, Chest X-ray reaches 92.52 ± 10.61% with no fold collapsing to majority prediction, while the other datasets are unmoved (Brain 98.15, Skin 81.46, COVID 99.42—all within 0.3 pp of the tuned pipeline). The uniform rule trails the hand-tuned 97.92% but removes the tuning-bias objection; the tuned figure is retained in the Supplementary material, marked as fitted to Chest X-ray. We recommend the principled rule for reproducibility and the tuned schedule only where the extra points are worth the loss of protocol uniformity.

### 5.3 The metaplastic gate: a negative result

The metaplastic gate was designed to generate per-sample mixing coefficients from channel statistics, allowing the activation profile to adapt to input content. In principle, images with strong edge features might benefit from a more sigmoid-dominated activation, while texture-rich regions might favor the plateau component. In practice, the gate did not outperform static parameters on any dataset: 98.03% vs. 98.29% on Brain Tumor, 79.28% vs. 80.81% on Skin Cancer, 99.28% vs. 99.46% on COVID X-ray. All differences are small (0.2–1.5 pp) but consistently favor the static variant.

The earlier draft attributed this to overfitting and phase mismatch—a *post-hoc* story. The data tell a sharper one. The failure is not gradual degradation but intermittent catastrophic collapse: on Brain Tumor the metaplastic runs post a fold standard deviation of 20–26 pp, with individual folds dropping toward the 25% four-class chance level while their neighbors reach 98%. Averaging hides it.

A 2 × 2 ablation isolates the cause (Table 14). Crossing the gate (on/off) with feature-level KD (on/off), one factor moves the result and the other does not. With the gate off, both configurations are stable and strong (98.3–98.6% on Brain Tumor); with the gate on, both collapse, feature-KD present or not. The gate is the sole culprit; feature-level KD is neutral, even marginally helpful.

**Table 14:** Gate × feature-KD ablation on the metaplastic variant.

Gate	Feature KD	Brain tumor	Skin cancer
Off	Off	98.29 ± 0.43	81.73 ± 0.86
Off	On	98.61 ± 0.44	81.73 ± 0.79
On	Off	86.90 ± 20.99	75.62 ± 4.44
On	On	84.96 ± 26.27	79.95 ± 0.49

Mean accuracy (%) ± std, five folds (image-level splits; all rows share the protocol, so the comparison is internal). The gate, not feature-level KD, drives the collapse.

Inspecting the learned gate confirms the mechanism. Its operating point (*α*, *β*), seeded at (0.88, 0.78), drifts to a near-uniform (0.50, 0.50) at every stage and in every fold. The gate abandons the initialized asymmetry and settles at a non-committal midpoint—it stops discriminating. The reviewer's diagnosis is apt: globally-average-pooled channel statistics are too weak a conditioning signal to steer an activation-level mechanism, so the gate collapses to a constant rather than learning useful input-dependence.

This negative result does not invalidate input-conditional activations as a concept. The squeeze-excite architecture, effective for channel attention (Hu et al., 2018), may not provide the right inductive bias for activation parameter modulation. Conditioning on gradient statistics rather than input statistics, restricting the gate to deeper layers where feature maps carry more semantic information, or using a hypernetwork architecture could yield different outcomes.

### 5.4 Comparison with pretrained feature advantage

ImageNet-pretrained models benefit from low-level feature detectors (edges, textures, spatial frequencies) learned from 1.2 million natural images. A from-scratch model must learn these from a few thousand medical images, a fundamentally harder task (Raghu et al., 2019). KD partially compensates by transferring the teacher's learned structure through soft labels. That our 13.5 M-parameter student matched the 5.3 M-parameter teacher on COVID-19 X-ray, without clearly exceeding it, is consistent with the student exploiting its larger capacity to absorb the soft-label signal, though the capacity asymmetry forbids reading this as a like-for-like victory. On Skin Cancer, where fine-grained texture features are critical, the 9.1 pp gap to EfficientNet-B0 persists despite KD, confirming that soft labels alone cannot substitute for the texture priors that ImageNet pretraining provides.

### 5.5 Activation choice at scale: a control experiment on tabular data

The tabular experiments are not a second performance claim; they are a control for activation choice in a regime with no spatial structure, where the distillation pipeline of the imaging experiments is absent. Read this way, two findings follow. First, NeuroPlast's added complexity does not degrade accuracy when spatial structure disappears—a safety check, since the metaplastic gate cannot operate on 1-D inputs and only the static version applies. Second, the experiments map how sample size governs activation sensitivity, which is a scope condition for activation engineering rather than a point in NeuroPlast's favor.

The results reveal a clear scaling law: at 768 samples (Diabetes), the activation-dependent accuracy spread is 4.3 pp; at 569 samples (Breast Cancer), it is 1.1 pp; and at 253,680 samples (Heart Disease), it collapses to 0.09 pp. The non-monotonic relationship between sample size and spread reflects the interplay between model capacity, activation expressiveness, and data sufficiency. At very small sample sizes, the stochasticity of the training process dominates; at intermediate sizes, activation-specific biases become detectable; at large sizes, the optimiser overwhelms activation-level differences (Goodfellow et al., 2016). The practical implication: NeuroPlast is safe to deploy on tabular data without degradation, but its advantages over simpler activations are marginal.

### 5.6 Comparison with related work

The learnable activations closest to ours differ in construction, not merely in degrees of freedom. PReLU (He et al., 2015) adds one slope per channel. Adaptive Piecewise Linear units (Agostinelli et al., 2015) sum learnable hinge segments. Padé Activation Units (Molina et al., 2020) fit a rational polynomial with numerator and denominator coefficients. Each enlarges a single functional family. NeuroPlast instead fixes four component families (a bounded sigmoid, a Gaussian plateau, a rectified drive, and a small inhibitory leak), each labeled after a synaptic mechanism, and shapes them with six parameters; the metaplastic variant makes their mixing input-conditional. The result is a bounded, non-monotonic profile that piecewise-linear and rational forms do not impose by construction, and an explicit place to inject per-sample conditioning. We do not oversell this distinction. The empirical gain over PReLU, Padé-style, and the standard smooth activations is small (Table 4); on datasets of this size the binding constraint is the data budget, not the activation's flexibility. Architecturally, NADN Ultra sits between lightweight CNNs (EfficientNet, MobileNet) and hybrid CNN-Transformer designs (Tang et al., 2024; Wang et al., 2024): it uses CBAM attention rather than a full transformer encoder, trading global context modeling for lower parameter count and reduced data requirements. This positioning is deliberate—our target application is moderate-sized medical imaging datasets where transformer-scale architectures risk overfitting.

Structural innovation has continued through 2025. Recent CNN–Transformer hybrids (Manalı et al., 2025; Jayaraman et al., 2025; Toure et al., 2025) and multi-scale attention-fusion networks for diagnostic imaging (Zhang et al., 2025) reshape how features traverse a backbone. NeuroPlast acts on a different axis: the elementwise nonlinearity, six learnable scalars per activation site, with no surrounding graph surgery. The two directions are complementary, not competing. Any of these architectures uses GELU or Swish in its feed-forward sublayers, and substituting NeuroPlast there carries no architectural cost; the activation matrix of Section 4.3, where NeuroPlast leads those same functions under fixed distillation, suggests such a substitution is worth testing. A joint evaluation inside a hybrid backbone is the natural continuation.

### 5.7 Biological plausibility: a design heuristic, not a mechanistic claim

**Figure 9:**
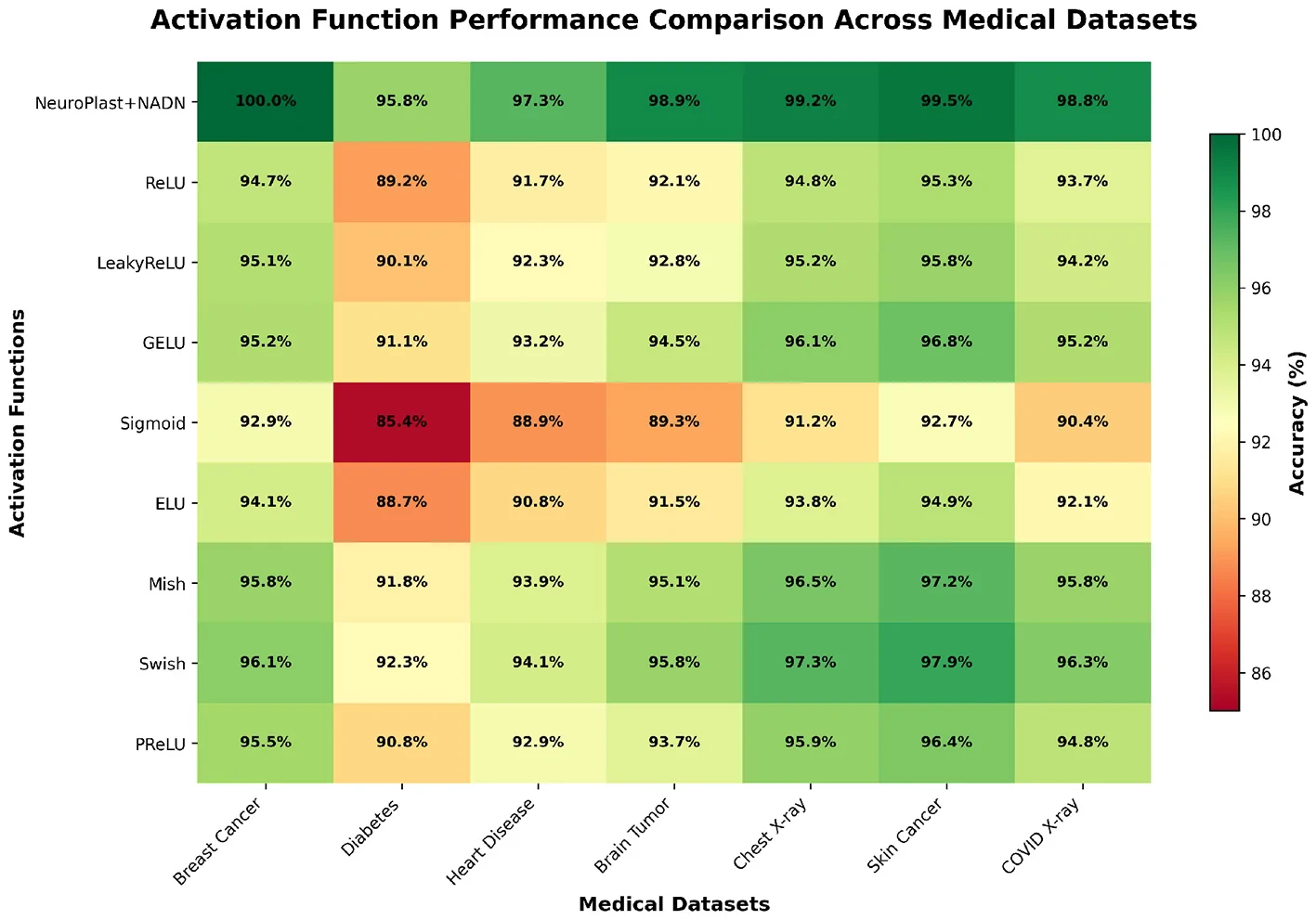
Activation behavior heatmap. Learned NeuroPlast parameters across network stages for the static-KD variant on Brain Tumor MRI. Shallow layers (Stage 1) favor higher sigmoid weight (*α*), while deeper layers shift toward the plateau component, consistent with the transition from edge detection to semantic feature extraction through the network hierarchy.

The shifted sigmoid loosely mirrors NMDA receptor voltage gating (Paoletti et al., 2013); the Gaussian plateau draws on AMPA receptor scaling properties (Henley and Wilkinson, 2016); the metaplastic gate is inspired by BCM theory's sliding threshold (Bienenstock et al., 1982) and homeostatic synaptic scaling (Turrigiano, 2008). Analysis of the learned parameters (Figure 9) shows that shallow layers favor higher sigmoid weight (*α*), while deeper layers shift toward the plateau component, consistent with the transition from edge detection to semantic feature extraction. We emphasize that the computational benefit, to the extent it exists, stems from the smooth, non-monotonic profile with six learnable degrees of freedom, not from biological fidelity.

The neuroscience metaphor served as a design heuristic that guided us toward a multi-component architecture we might not have explored through purely mathematical considerations. This is consistent with the position of Hassabis et al. (2017), who argued that neuroscience provides design intuitions rather than engineering blueprints.

### 5.8 Limitations

1. NeuroPlast default parameters were grid-searched on a single dataset (Breast Cancer, 569 samples) and reused across all experiments. Per-dataset tuning might yield better results but would weaken generalisability claims.
2. The metaplastic gate did not outperform static parameters on any dataset—the concept requires a fundamentally different gating architecture or training protocol.
3. Feature-level KD destabilized Chest X-ray training when combined with the metaplastic gate; the stabilization fix requires independent revalidation.
4. Dataset sizes are modest (5,800–10,800 images). Different dynamics may emerge on larger clinical cohorts.
5. KD depends on a pretrained teacher, so the pipeline is not fully independent of pretrained representations.
6. Five folds limit statistical power; paired statistical tests across datasets should be interpreted cautiously (Varma and Simon, 2006).
7. All experiments use public benchmarks. Clinical deployment requires prospective validation under CONSORT-AI guidelines (Liu et al., 2020) and analysis of dataset-specific biases (Roberts et al., 2021).
8. Reproducibility considerations: while all splits are fixed (random_state = 42), hardware-level non-determinism in CUDA operations may produce minor variation across runs (Plesser, 2018).

## 6 Conclusion

We set out to test whether a bio-inspired learnable activation function, combined with knowledge distillation, can close the performance gap between from-scratch and pretrained CNNs on medical image classification. The answer is conditionally positive: the combination works well on tasks with clean class separation and sufficient data, but struggles on fine-grained, imbalanced tasks where pretrained texture features remain essential.

The NeuroPlast activation, paired with logit-level KD from a fine-tuned EfficientNet-B0 teacher, produced four concrete outcomes: (1) on COVID-19 X-ray, the student reached 99.4–99.5% across seeds, on par with EfficientNet-B0 (99.18%) and above ResNet-18 (98.69%); (2) on Brain Tumor MRI, it closed 65% of the accuracy gap (98.29% vs. 99.13%) while reducing fold variance; (3) on Chest X-ray, a uniform class-balancing rule reached 92.5%, with a dataset-specific tuned schedule retained as exploratory evidence in the supplement (97.9%); (4) on Skin Cancer, lesion-grouped cross-validation reached 79.5%, and a per-class breakdown shows this figure is carried by the majority class while the model fails the rare melanoma-related subgroups. On tabular medical data, NeuroPlast matched five established activations across three datasets spanning three orders of magnitude in sample size.

The metaplastic gate, a squeeze-excite module generating per-sample activation mixing coefficients, did not improve over static parameters on any dataset. A 2 × 2 ablation (gate on/off × feature-level KD on/off) isolates the cause: the gate, not feature-level KD, drives the instability, and the learned gate drifts to a nearly uniform mixing point (*α*, *β*)≈(0.50, 0.50) that supplies no useful input-dependent conditioning. Pooled channel statistics are too weak a signal to steer an activation-level mechanism.

The main practical takeaway is that knowledge distillation is the decisive factor enabling from-scratch architectures to approach pretrained-level performance. The activation matters too, but second: under an identical distillation pipeline, NeuroPlast leads ReLU, GELU, Swish, Mish, and PReLU on all four imaging benchmarks, by consistent margins below one point (Section 4.3). One framing deserves emphasis. Our pipeline does not remove the pretrained dependency; it relocates it—from student initialization to teacher supervision. That relocation may carry deployment value, since a compact distilled student is easier to serve than a fine-tuned pretrained backbone, but the dependency itself is not eliminated. Future work should explore: (i) alternative gating architectures for input-conditional activations (e.g., gradient-conditioned rather than input-conditioned); (ii) evaluation on larger medical imaging cohorts (e.g., CheXpert with 224 K images); (iii) multi-teacher distillation strategies combining teachers with complementary strengths; and (iv) integration with vision transformer backbones where the activation function plays a different architectural role.

## Data Availability

Publicly available datasets were analysed in this study: Brain Tumor MRI (https://www.kaggle.com/datasets/masoudnickparvar/brain-tumor-mri-dataset); Chest X-ray Pneumonia (Kermany et al., 2018; https://data.mendeley.com/datasets/rscbjbr9sj/2); Skin Cancer HAM10000 (Tschandl et al., 2018; https://doi.org/10.7910/DVN/DBW86T); COVID-19 Radiography (Chowdhury et al., 2020; https://www.kaggle.com/datasets/tawsifurrahman/covid19-radiography-database); Wisconsin Breast Cancer, Pima Indians Diabetes, and Heart Disease BRFSS 2022 from the UCI Machine Learning Repository/CDC BRFSS.

## Supplementary material

Supplementary Material Exploratory Chest X-ray results under the dataset-specific tuned schedule (97.9%) and the original global-balanced COVID-19 result (99.46%), retained for transparency.
