# Supplementary material for: NeuroPlast: a learnable activation function evaluated under knowledge distillation for medical image classification
Source: Front Artif Intell. 2026 Aug 31;9:1849571. doi: 10.3389/frai.2026.1849571 (PMC13572696; doi:10.3389/frai.2026.1849571)
Supplement: Supplementary file 1 [file Data_Sheet_1.PDF]

Supplementary Material

NeuroPlast: A Learnable Activation Function Evaluated under Knowledge Distillation for Medical Image Classification

Vaussenat, Bhattacharya, Payette, Desmettre, Saidi, Gagnon, Cloutier

This document retains two results referenced in the main text: the original COVID-19 balancing outcome (Section 4.7) and the tuned Chest X-ray stabilisation protocol (Section 5.3). Both are kept for transparency and reproducibility of the original submission; the main text reports the corrected, protocol-uniform figures.

S1. COVID-19: original global-balanced result

The first submission balanced COVID-19 by majority-class subsampling applied globally, before fold creation. This lets the validation distribution influence the balancing decision, and is corrected in the main text by moving the operation inside each training fold (main text 99.54 % ± 0.12 %). The original global-balanced static-KD result is retained here.

Protocol	Accuracy (%)	Where reported
Global balancing (before folds)	99.46 ± 0.15	Original submission (this table)
Within-fold balancing (corrected)	99.54 ± 0.12	Main text, Section 4.7

The corrected protocol does not degrade the result; the two figures agree within fold noise.

S2. Chest X-ray: tuned stabilisation recipe

The original Chest X-ray protocol used a dataset-specific stabilisation recipe, selected after observing fold-level collapse under the standard pipeline. Because that schedule was fitted to one dataset's validation behaviour, it introduces tuning bias relative to the uniform protocol used on the other three datasets. The main text replaces it with a single a-priori rule (inverse-frequency class weighting applied identically to all datasets; 92.52 % ± 10.61 % on Chest X-ray, no fold collapsing). The tuned recipe and its result are retained here for reproducibility of the original submission.

Tuned recipe (Chest X-ray only).

1. 20 epochs of pure task-loss training before any KD signal.
2. Gradual KD ramp-in over epochs 20–25.
3. Fixed KD weight $\alpha = 0.5$ (rather than 0.3), to maintain stronger task supervision.
4. Label smoothing disabled.
5. Augmentation disabled until epoch 25.

**Chest X-ray protocol**	**Accuracy (%)**	**Note**
Tuned recipe (this supplement)	97.92 ± 0.27	Fitted to Chest X-ray (tuning bias)
Uniform class-balanced rule	92.52 ± 10.61	Main text, Section 5.3

The uniform rule trails the tuned recipe but removes the tuning-bias objection and preserves protocol uniformity across all four datasets.
